# Low fertility awareness and associated factors - a multi-centre cross-sectional study among abortion-seeking women in Sweden

**DOI:** 10.1186/s40834-025-00401-3

**Published:** 2025-09-17

**Authors:** Sara Holmdahl Arciniegas, Marlene Makenzius

**Affiliations:** 1 Department of Health Sciences, Mid Sweden University, Kunskapens väg 8, Östersund, 83145 Sweden; 2 Department of Global Public Health, Karolinska institutet, Stockholm, Sweden; 3 Department of Women’s and Children’s Health, Karolinska institutet, Stockholm, Sweden

**Keywords:** Contraception, Fertility awareness, Induced abortion, Reproductive health, Unintended pregnancy, Unwanted pregnancy

## Abstract

**Background:**

The Swedish model for contraceptive counselling is well developed, with free and easily accessible services, and contraceptives that are free or subsidised. Despite this, many women have an unmet need for contraception.

**Aim:**

The aim of this study was to investigate the reasons for not using contraceptive methods at the time of conception among abortion-seeking women, and to identify factors associated with unawareness of their potential to become pregnant at that time.

**Methods:**

A multi-centre cross-sectional survey was conducted among 623 abortion-seeking women at seven family planning clinics in Sweden in 2021. The questionnaire comprised 39 items, one of which explicitly addressed the reasons for not using contraception at the time of conception. Bivariate and multivariate analyses were conducted using odds ratios (ORs) and confidence intervals (CIs).

**Results:**

Of the 623 women, 387 reported not using contraception at the time of conception. The most common reason was unawareness of their potential to become pregnant at that time, considered as low fertility awareness (LFA), reported by 142 (37%) women. No significant sociodemographic or lifestyle differences were found between women with LFA and those reporting other reasons for not using contraception. However, LFA was more common among women who had not used contraception in the previous 12 months (OR = 2.912, CI: 1.844–4.601), and previous abortion experience was a protective factor (OR = 0.617, CI: 0.398–0.958). A significantly lower proportion of women reported contraceptive use at the time of conception compared to the preceding year.

**Conclusions:**

LFA is common and linked to no contraceptive use in the previous year and no prior abortion history, and contraceptive use at conception is lower than over the preceding year indicating gaps in continuity and adherence. These findings support service models that turn counselling into timely initiation with verified uptake and sustained use.

**Supplementary Information:**

The online version contains supplementary material available at 10.1186/s40834-025-00401-3.

## Introduction

The United Nations Sustainable Development Goals for 2030 include two targets related to reproductive health [1]. Goal 3.7 aims to ensure universal access to sexual and reproductive healthcare services, including family planning, education, and the integration of reproductive health into national strategies and programmes. Goal 5.6 seeks to ensure universal access to sexual and reproductive health and rights [1]. Similar to Agenda 2030, the Public Health Agency of Sweden prioritizes sexual and reproductive health and rights as part of its broader commitment to health equity [2].

Unintended pregnancy and abortion rates vary globally. Low-income countries report approximately 38 abortions per 1,000 women of reproductive age; middle-income countries, 44 per 1,000; and high-income countries, 15 per 1,000 [3]. In Sweden, the 2021 rate was 18 per 1,000—highest among Nordic countries [4].

Abortion is legal upon a woman’s request until the 18th week of pregnancy. Beyond that, approval from the National Board of Health and Welfare is required [5]. Common reasons for abortion in Sweden include economic constraints, relationship timing, work or study priorities, relationship uncertainty, and age [6].

Currently in Sweden, long-acting reversible contraception (LARC) methods such as intrauterine devices (IUDs), implants, injectables, and oral contraceptives are free for youth up to age 25 and heavily subsidized beyond that. Contraceptive counselling including provision of emergency contraception (EC), are primarily provided (for free) by midwives at maternal health clinics, youth clinics abortion clinics and district healthcare centres, with the option to contact the national helpline for guidance or to book appointments [7]. EC can also be bought (subsidized) at pharmacies without prescription.

Despite a range of available methods, unmet contraceptive needs persist. In Western countries, common reasons for rejecting hormonal methods include concerns about side effects, libido, future fertility and gender concerns [8, 9]. In Sweden, although contraceptive counselling by midwives is a cornerstone of care, unmet contraceptive needs have grown [10].

Fertility awareness (FA) refers to knowledge of the fertile window, based on natural body signs such as cervical fluid and basal body temperature. Low fertility awareness (LFA) is common among both women and men, even among those attempting to conceive [11–13].

Misunderstandings about ovulation timing, combined with hesitance around hormonal contraception, contribute to LFA. Ovulation varies widely, complicating fertility prediction [14].

Comprehensive sexuality education (CSE) has been mandatory in Swedish schools since 1955 and includes topics related to sexual health, equality, and diversity [15]. Despite this, a gap remains between access to contraception and its effective use.

## Aim

The aim of this study was to investigate the reasons for not using contraceptive methods at the time of conception among abortion-seeking women, and to identify factors associated with unawareness of their potential to become pregnant at that time.

## Methods

This sub-study was part of a larger multi-centre cross-sectional study on abortion and contraceptive use during the COVID-19 pandemic [16]. The study followed the STROBE checklist for cross-sectional studies [17].

From January to June 2021, data were collected at seven family planning clinics in urban and suburban Sweden. All clinics performed abortions and were located in areas covering diverse socioeconomic contexts. Several clinics served large catchment areas that included multiple municipalities with varying socioeconomic profiles.

Women seeking induced abortion received an information leaflet; those who agreed filled out an anonymous paper questionnaire, returned to staff. Return of the questionnaire constituted informed consent.

The questionnaire, originally developed by Larsson et al. [18], was revised in 2009 [6] and updated in 2021 [16] with pandemic-related questions. It included 39 items covering sociodemographic data, health, pregnancies, abortions, contraceptive use, and counselling access during the pandemic. One question asked about reasons for not using contraception at the time of conception, analysed in the current study. The question had with 16 predefined options and one open-ended response. Multiple answers were permitted. Respondents were divided into two groups: those who stated they did not know they could become pregnant at the time of conception (LFA group, *n* = 142), and all others (*n* = 481), referring to women who reported other reasons for not using contraception. Bivariate analyses were performed to assess associations between the dependent variable (LFA) and each independent variable separately. Pearson’s chi-square test was used for categorical variables, or Fisher’s exact test when expected cell counts were below five. Independent-samples t-tests were applied for continuous variables. Variables included in the analysis were age, country of birth, occupation, education, relationship status, number of children, abortion history, contraceptive use in the previous 12 months, and tobacco use. Odds ratios (OR) and 95% confidence intervals (CI) were calculated for categorical variables. Variables with statistically significant associations in the bivariate analyses were included in a multivariate logistic regression model to identify independent predictors of LFA. Some variables were dichotomised due to small sample sizes. A *p*-value of < 0.05 was considered statistically significant. All analyses were conducted using IBM SPSS Statistics version 29.

## Results

43% of respondents were not using contraception at the time of conception, and 25.7% had not used any contraceptive method during the last year, highlighting gaps in consistent use (Table 1).

**Table 1:** Reported use of contraceptive methods at the time of conception the last 12 months (*n* = 614)

Contraceptive method	At Conception *n* (%)	Last 12 Months *n* (%)
**No method**	**268 (43)**	**160 (25.7)**
Condom	124 (19.9)	228 (36.6)
Withdrawal	110 (17.7)	152 (24.4)
Oral contraceptive pill	86 (13.8)	196 (31.5)
Calendar method	56 (9)	71 (11.4)
App (e.g. Natural cycles)	37 (5.9)	63 (10.1)
Emergency contraceptive pill	36 (5.8)	81 (13)
Intrauterine device	9 (1.4)	53 (8.5)
Breast feeding	6 (1)	18 (2.9)
Contraceptive vaginal ring	3 (0.5)	11 (1.8)
Implant	3 (0.5)	35 (5.6)
Contraceptive patch	2 (0.3)	11 (1.8)
Diaphragm	1 (0.2)	1 (0.2)

Missing’s *n* = 9 (1.4%)

Table 2 shows the reported reasons; the most frequent was the belief they could not become pregnant (*n* = 142 of 387), followed by previous negative experiences with contraceptives (*n* = 118). Of the 387 respondents, 76 (19.6%) added open-ended comments, typically citing reluctance to use hormonal contraception, delays in accessing new contraception, or side effects.

**Table 2:** Reasons given for non-use of contraception at conception among those reporting non-use (*n* = 387); low fertility awareness group compared with other non-users (multiple responses permitted)

Reasons for not using any contraceptives	Total (*n* = 387) *n* (%)	Low fertility awareness (*n* = 142) *n* (%)	All other non-users (*n* = 245) *n* (%)
Did not know any method	5 (1.3)	0 (0)	5 (1.3)
Did not plan to have sex	73 (18.9)	20 (14.1)	73 (18.9)
Did not dare to suggest it	0 (-)	0 (-)	0 (-)
The partner did not want to	9 (2.3)	4 (2.8)	5 (1.0)
Was forced into sex	0 (-)	0 (-)	0 (-)
Been advised against it	2 (-)	1 (0.2)	1 (0.7)
Did not get a new prescription in time	18 (4.6)	7 (4.9)	11 (2.3)
Did not know where to obtain contraceptives	1 (-)	0 (-)	1 (-)
Bad experiences with contraceptives	118 (30)	31 (21.8)	87 (18.1)
Thought I was sterile	15 (3.9)	9 (6.3)	6 (1.2)
Thought my partner was sterile	6 (1.6)	2 (1.4)	4 (0.8)
Too expensive	8 (2.1)	0 (-)	8 (1.7)
Took the risk	66 (17.1)	19 (13.4)	47 (9.8)
Under the influence of alcohol	9 (2.3)	3 (2.1)	6 (1.2)
Under the influence of drugs	2 (-)	0 (-)	2 (0.4)
Other reason	76 (19.6)	12 (8.5)	64 (13.3)

Table 3 compares the characteristics of women with LFA (*n* = 142) and others (*n* = 481). LFA was more common among those without a history of abortion and those who had not used contraception in the past year. It was less common among employed women.

**Table 3:** Characteristics of the abortion-seeking women

Characteristics		Total *n* = 623 *n* (%)						Low fertility awareness *n* = 142 *n* (%)					All others *n* = 481 *n* (%)		*P*-value	
**Age***n* = 618 (range 14–47 years)																
Mean ± SD		28.9 ± 6.6						29.2 ± 6.8					28.8 ± 6.5		.573^a^	
–24		174 (27.9)						38 (26.8)					136 (28.3)			
25–29		152 (24.4)						37 (26.1)					115 (23.9)			
30–34		164 (26.3)						39 (27.5)					125 (26)			
35+		128 (20.5)						28 (19.7)					100 (20.8)			
Missing		5 (0.8)						0					5 (1)			
**Country of birth***n* = 613																
Sweden		526 (84.4)						120 (84.5)					406 (84.4)		.871^b^	
Other Nordic countries		6 (1)						1 (0.7)					5 (1)			
Outside the Nordic countries		81 (13)						20 (14.1)					61 (12.7)			
Missing		10 (1.6)						1 (0.7)					9 (1.9)			
**Occupation***n* = 618																
Student		128 (20.5)						30 (21.1)					98 (20.4)		.025^b^	
Employed		398 (63.9)						85 (59.9)					313 (65.1)			
Unemployed		39 (6.3)						7 (4.9)					32 (6.7)			
Housewife/maternity leave		43 (6.9)						14 (9.9)					29 (6)			
Other		10 (1.6)						6 (4.2)					4 (0.8)			
Missing		5 (0.8)						0					5 (1)			
**Education level***n* = 616																
Comprehensive school (9 years)		75 (12)						19 (13.4)					56 (11.6)		.842^b^	
Upper secondary school (12 years)		364 (58.4)						81 (57)					283 (58.8)			
University		177 (28.4)						41 (28.9)					136 (28.3)			
Missing		7 (1.1)						1 (0.7)					6 (1.2)			
**In a steady relationship ***n* = 614																
No		137 (22)						31 (21.8)					106 (22)		1.000^c^	
Yes		477 (76.6)						109 (76.8)					368 (76.5)			
Missing		9 (1.4)						2 (1.4)					7 (1.5)			
**Previous children***n* = 581																
0	271 (43.5)			69 (48.6)	202 (42)					.282^c^						
1/+	310 (49.8)			67 (47.1)	243 (50.5)											
Missing	42 (6.7)			6 (4.2)	36 (7.5)											
**Previous induced abortions***n* = 581																
0		279 (44.8)					76 (53.5)					203 (42.2)			.040^c^	
1/+		302 (48.5)					60 (42.2)					242 (50.3)				
Missing		42 (6.7)					6 (4.2)					36 (7.5)				
**Contraceptive use during the past 12 months***n* = 616																
Had used contraception for the last 12 months	456 (73.2)			80 (56.3)	376 (78.2)				< .001^c^							
No method used in the last 12 months	160 (25.7)			62 (43.7)	98 (20.4)											
Missing	7 (1.1)			-	7 (1.5)											
**Planning to use a contraceptive method after the abortion***n* = 600																
No			23 (3.7)			5 (3.5)					18 (3.7)					.727^b^
Yes			527 (84.6)			122 (85.9)					405 (84.2)					
Do not know			50 (8)			14 (9.9)					36 (7.5)					
Missing			23 (3.7)			1 (0.7)					22 (4.6)					
**Tobacco use***n* = 613																
No			351 (56.3)			85 (59.9)					266 (55.3)					.499^c^
Smoke or/and snuff use			262 (42.1)			57 (40)					205 (42.6)					
Missing			10 (1.6)			-					10 (2.1)					

^a^ Independent samples t-test

^b^ Pearson Chi-square, 2 sided

^c^ Fisher’s exact test, 2 sided

Table 4 shows that lack of contraceptive use in the previous 12 months (OR = 2.91, CI: 1.84–4.6) and no previous abortion (OR = 0.62, CI: 0.4–0.96) were significantly associated with LFA.

**Table 4:** Independent factors associated with low fertility awareness (*n* = 578)

Factors	OR	CI 95%		*P*-value
		Lower	Upper	
No method used during the past 12 months Ref: Use of any contraceptive method during the past 12 months	2.91	1.84	4.6	< 0.001
≥ 1 previous abortion Ref: 0 previous abortion	0.62	0.4	0.96	0.032
Employed Ref: Unemployed	1.06	0.64	1.8	0.823

OR = Odds ratio; ci = confidence interval

## Discussion

The study found that the primary reason for not using contraception was the belief that conception was not possible at the time. LFA was significantly associated with no contraceptive use in the prior year and no history of induced abortion. Other factors, such as age, education, socioeconomic status, or number of children, showed no significant associations. This is consistent with previous research showing that LFA is not necessarily linked to age or education [12, 19]. While some studies show a correlation between education and FA [11], results remain mixed.

The association between LFA and non-use of contraception highlights how misconceptions shape contraceptive behaviour, consistent with prior studies [20, 21] citing low perceived pregnancy risk and access barriers.

During the COVID-19 pandemic in Sweden, access to contraceptive counselling remained largely unaffected, with a national study reporting that almost 70% of women aged 16–49 experienced access “as usual” and 14% utilised telemedicine services. Satisfaction with counselling was high [22], suggesting that pandemic-related service disruptions are unlikely to explain trends in multiple abortions.

According to a national report, approximately 37,000 induced abortions are performed annually, and in 2023, 45% of those seeking abortion had undergone at least one previous procedure [23]. Similar proportions have been observed in other high-income countries, supporting the notion that repeat abortions are a persistent public health concern and emphasising the need for robust post-abortion contraceptive counselling [6, 24, 25].

A key result was the gap between contraceptive use in the previous year and use at the time of conception. Many women had used contraception at some point, yet were not using a method when they became pregnant. This suggests discontinuation, inconsistent use, or a mismatch between chosen methods and everyday life. Short-acting, user-dependent methods (pills, condoms, withdrawal) were much less common at the time of conception than over the past year, which is consistent with their higher real-world failure when use is imperfect [26]. In contrast, LARCs (IUDs, implants) were under-represented at the time of conception, which fits with their high effectiveness and/or lower uptake.

Evidence from other European settings provides useful context. In a French multicentre study of women with repeated abortions, 78.8% changed to a different method after the abortion. Among those who changed, 26.2% were prescribed a LARC but only 51.8% of them were using it at follow-up [27]. This pattern shows how easily intentions and prescriptions can fail to translate into real use. A European survey of simulated consultations also reported missed opportunities in counselling based on differences in timing, content, and how well advice fits women’s preferences [28]. Historical and sociocultural analyses that medical traditions and gendered expectations can maintain reliance on short-acting, user-dependent methods even when more effective options are available [29].

Method-specific failure rates may help explain the current findings. French population estimates show very low first-year failure for IUDs (~ 1.1%) and much higher failure for user-dependent methods (e.g., withdrawal ~ 10.1%; fertility awareness ~ 7.7%) [30]. It is therefore unsurprising that short-acting methods and family planning feature more often at conception in the current sample, while LARC users are less likely to appear among unintended pregnancies.

Finally, policy and routine contexts matters. Sweden provides easy access to contraceptive counselling and free or subsidised contraception, including EC. Such policies vary across the EU. Although these policies help, they are not sufficient on their own [31, 32]. A study from Finland found that verified immediate LARC initiation at the time of medical abortion was linked to markedly lower risk of a subsequent abortion; merely intending to start a LARC later did not show the same benefit [33]. This reinforces the interpretation that outcomes depend not only on counselling but also on timing, logistics, and completion of initiation.

## Strengths and limitations

A strength of this study is its broad geographical representation. However, approximately one-third of eligible women were not invited, often due to language barriers, which may have introduced selection bias and affected the results. Immigrant women, who often have lower contraceptive use and higher abortion rates [34], were underrepresented. The study was not originally designed to examine LFA, which may limit the validity and reliability of how this construct was measured. In addition, the exclusion of men, despite their important role in reproductive outcomes [35], reduces the broader applicability of the findings. Therefore, LFA as a distinct concept remains uncertain and should be explored further in future research.

## Implications

The high rate of non-use of contraceptive at conception despite prior use signals a need to shift from simply promoting access to encouraging correct and continuous use. Targeted counselling and cost-free access (like in Sweden), although important but may not be sufficient on their own to increase LARC use. Additional interventions, such as provider training and rapid access to insertion, further improved LARC uptake in clinical settings [33, 36]. International examples from Finland and Australia support the combination of cost removal and provider incentives or fast-track access as effective strategies to boost LARC use and reduce abortion rates [33, 36, 37].

In addition, since pills, condoms, and withdrawal continue to dominate usage patterns, education should focus on correct use, backup strategies, and transitions to more effective options. Efforts to increase use must go beyond availability and address individual concerns, provider engagement, and the timing of counselling, particularly in relation to life events such as abortion or childbirth. Some individuals may stop or change methods due to (fear of) side effects, misconceptions, partner issues, lack of awareness or lifestyle changes. Health systems should provide supportive follow-up to prevent method gaps, including proactive rescheduling and digital tools for reminders.

## Conclusion

This study points to two priorities. First, low fertility awareness is common and associated with no contraceptive use in the previous year and no prior abortion history, indicating that knowledge and experience shape behaviour; fertility awareness should therefore be strengthened and include men. Second, the markedly lower use of contraception at conception than over the preceding year reveals a gap in continuity and adherence, and a mismatch between method and user. Together, these findings support service models that convert counselling into timely initiation with verified uptake and provide support for sustained use.

## Supplementary Information

Below is the link to the electronic supplementary material.

Supplementary Material 1

## Data Availability

The datasets used and/or analysed during the current study are available from the corresponding author on reasonable request.
