# Supplementary material for: Low fertility awareness and associated factors - a multi-centre cross-sectional study among abortion-seeking women in Sweden
Source: Contracept Reprod Med. 2025 Sep 17;10:55. doi: 10.1186/s40834-025-00401-3 (PMC12442278; doi:10.1186/s40834-025-00401-3)
Supplement: Supplementary file 1 — Supplementary Material 1 [file 40834_2025_401_MOESM1_ESM.docx]

|  |
| --- |
| Abortions and contraception in times of the Covid-19 pandemic |
| Questionnaire for those who are seeking an abortion |

|  |
| --- |

**
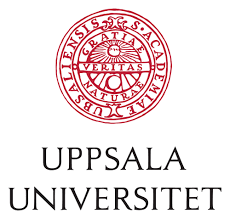
**

| 1. How old are you? ................................ | | | |  | |
| --- | --- | --- | --- | --- | --- |
| 1. In which country were you born? | | | | Sweden  Another country  Which?................................ | |
| 1. In which country were your parents born? | | | | Both are born in Sweden    Mother is born in .............................................  Father is born in ............................................... | |
| 1. What is your current main occupation? (Put a cross) | | | | | |
|  | Studying  Working  Looking for work  Working at home/parental leave    Other................................................................................... | | | | |
| 1. What is the highest education you completed? | | | | Primary school or equivalent  High School  University/college | |
| 1. Do you have a steady relationship/permanent partner? | | | | No  Yes  If yes – how long?    ................. years ................... months | |
| 1. Have you been pregnant before? | | | No  Yes  Number of births........... Abortions ....................  Miscarriages............... Ectopic Pregnancies................... | | |
| 1. Do you smoke? | | | | | |
| No | | Yes, sometimes | | | Yes, daily |
| 1. Do you use snuff? | | | | | |
| No | | Yes, sometimes | | | Yes, daily |
| 1. Have you consumed any alcohol in the last 3 months? | | | | | |
| No | | Yes, sometimes | | | Yes, daily |
| 1. Do you use drugs? | | | | | |
| No | | Yes, sometimes | | | Yes, daily |
| If yes, which one/ones? ........................................................................................................... | | | | | |

| 1. How do you feel about making the decisions about the abortion? (Tick one of the boxes) | | | | | | | | | | | |
| --- | --- | --- | --- | --- | --- | --- | --- | --- | --- | --- | --- |
| 1  Very easy | 2 | | 3 | | 4 | 5 | | | 6 | | 7  Very difficult |
| 1. Did you discuss the abortion decision with someone before the visit today? | | | | | | | No  Yes | | | | |
| If yes – who? ..........................................................................................................................................  .................................................................................................................................................................................... | | | | | | | | | | | |
| 1. State your main reason for the abortion. (Check as many circles as you need) | | | | | | | | | | | |
| ◯ Physical illness  ◯ Am single  ◯ Uncertain about relationship to partner  ◯ Too early in the relationship  ◯ Want to study first  ◯ Want to get married first  ◯ Victim of domestic violence | | ◯ Possible health problem affecting foetus  ◯ Spacing between children  ◯ Family completed  ◯ My partner is hesitant  ◯ Want to work first  ◯ My parents don’t want me to have a baby  ◯ Don’t want to bring children into this world | | | | | | | | ◯ Mental health  ◯ Poor economy  ◯ Too young  ◯ Too old  ◯ Unsuitable housing  ◯ Afraid to give birth  ◯ Covid-19 | |
| ◯ Other ................................................................................................................................................... | | | | | | | | | | | |
| 1. Which contraceptive method(s) have you used in the last year?   (Check as many circles as you need). | | | | | | | | | | | |
| ◯ No method  ◯ App (e.g. Natural Cycles)  ◯ IUD/hormonal IUD  ◯ Birth control patch  ◯ Breastfeeding | | | | ◯ Interrupted intercourse  ◯ Condom  ◯ Birth control pills  ◯ Emergency contraceptive pills | | | | ◯ Safe periods  ◯ Pessary  ◯ P-ring  ◯ P-rod | | | |
| ◯ Other..................................................................................................................................................... | | | | | | | | | | | |
| 1. Have you visited a clinic for contraceptive advice in the last year? | | | | | | | No  Yes | | | | |
| If yes – which one/s? ............................................................................................................................ | | | | | | | | | | | |
| 1. Have you changed your birth control method in the last year? | | | | | | | No  Yes | | | | |
| If yes – why? ....................................................................................................................................... | | | | | | | | | | | |
| 1. What contraceptive method did you use when you got pregnant? | | | | | | | | | | | |
| ◯ No method  ◯ App (e.g. Natural Cycles)  ◯ IUD/hormonal IUD  ◯ Birth control patch  ◯ Breastfeeding | | | | ◯ Interrupted intercourse  ◯ Condom  ◯ Birth control pills  ◯ Emergency contraceptive pills | | | | ◯ Safe periods  ◯ Pessary  ◯ P-ring  ◯ P-rod | | | |
| ◯ Other...................................................................................................................................................... | | | | | | | | | | | |

| 1. If you did not use any contraceptive method, what were your main reasons?   (Multiple answers possible). | | | | | | | |
| --- | --- | --- | --- | --- | --- | --- | --- |
| ◯ Didn’t know any method  ◯ Didn’t plan to have sex  ◯ Didn’t dare to suggest it  ◯ The partner didn’t want to  ◯ Was forced into sex  ◯ Been advised against it | | ◯ Did not get a new prescription in time  ◯ Didn’t know where to get contraceptives  ◯ Didn’t think I could get pregnant then  ◯ Bad experiences with contraceptives  ◯ Thought I was sterile  ◯ Thought my partner was sterile | | | | | ◯ Too expensive  ◯ Took the risk  ◯ Under the influence of alcohol  ◯ Under the influence of drugs |
| ◯ Other...................................................................................................................................................... | | | | | | | |
| 1. If you did not use hormonal contraceptives because of bad experiences, what were your main reasons? (Multiple answers possible). | | | | | | | |
| ◯ Mood swings  ◯ Depressed mood  ◯ Not comfortable with hormones | | ◯ Planned pregnancy  ◯ Weight gain | | | ◯ Decreased sex drive  ◯ Other side effects | | |
| ◯ Other...................................................................................................................................................... | | | | | | | |
| 1. If you used a birth control method, why didn't it work? | | | | | | | |
| Had miscounted the days  The App did not work  Condom broke/slipped off  Vomited or had diarrhoea | | | The intercourse was not interrupted  Forgot to change the birth control ring/birth control patch  Did not use a condom all the time  IUD did not work /had come out | | | | Forgot birth control pills  Forgot the pessary  Don’t know |
| Other...................................................................................................................................................... | | | | | | | |
| 1. If you choose to have an abortion, do you plan to use any contraceptive method after the abortion? | | | | | | | |
| No | | Yes | | | Don’t know | | |
| If yes – do you know which method, you plan to use? | | | | | | | |
|  | No | | | Yes | |  | |
| 1. If yes – which method, do you plan to use? | | | | | | | |
| ◯ No method  ◯ App (e.g. Natural Cycles)  ◯ IUD/hormonal IUD  ◯ Birth control patch | | ◯ Interrupted intercourse  ◯ Condom  ◯ Birth control pills  ◯ Emergency birth control pills | | | ◯ Safe periods  ◯ Pessary  ◯ P-ring  ◯ P-rod | | |
| ◯ Other...................................................................................................................................................... | | | | | | | |

| 1. Has the Covid-19 pandemic affected your decision about the abortion? | | | | | | |
| --- | --- | --- | --- | --- | --- | --- |
| No | | Yes, a little | | | Yes, a lot | |
| If yes, in what way? ...........................................................................................................................  ........................................................................................................................................................................ | | | | | | |
| 1. Since the pandemic broke out, have you had an abortion because of Covid-19? | | | | | | |
| No | | | | Yes | | |
| 1. Have you had symptoms of Covid-19 in the past year? | | | | | | |
| No | | | | Yes | | |
| 1. Have you been diagnosed with Covid-19 verified with a Covid test (e.g. PCR, antigen or antibodies)? | | | | | | |
| No | | | | Yes | | |
| 1. If you have had verified Covid-19, are you now symptom-free? | | | No  Yes | | | |
| If no - how many months have you had symptoms? ....................................................................... | | | | | | |
| 1. Has your partner had symptoms of Covid-19 in the past year? | | | | | | |
| No | | | | Yes | | |
| 1. Has your partner been diagnosed with Covid-19 verified with a Covid test (e.g. PCR, antigen or antibodies)? | | | | | | |
| No | | | | Yes | | |
| 1. Have you had difficulty finding time for contraceptive advice/prescription due to the Covid-19 pandemic? | | | | | | |
| No | | | | Yes | | |
| 1. Has it been because of: | Closed clinics  Own illness | | | | | Other reason |
| 1. As a result of that, have you: | | | | | | |
| 1. Changed contraceptive method? | | | | No  Yes | | |
| 1. Had an unplanned pregnancy? | | | | No  Yes | | |
| 1. If yes to the previous question - had an abortion? | | | | No  Yes | | |

| From 1 July 2018, the Consent Act was introduced in Sweden. According to the law, it is forbidden to have sex with a person who has not explicitly said yes or actively shown that they want to have sex. | | | | |
| --- | --- | --- | --- | --- |
| 1. Were you asked for consent, or actively indicated that you wanted to have sex, during the period when you became pregnant? | | | | |
| No | Don’t remember | | Yes | |
| 1. In the past 12 months, have you been exposed to: | | | | |
| Physical violence/abuse? | | No | | Yes |
| Mental ill treatment/abuse? | | No | | Yes |
| Sexual violence /abuse? | | No | | Yes |
| 1. How do you think your physical (bodily) well-being was: | | | | |
| 1. Before you knew you were pregnant? | | 1. After you found out you were pregnant? | | |
| □ Very good  □ Neither well nor poor  □ Sort of poor □ Very poor | | □ Very good  □ Neither well nor poor  □ Sort of poor □ Very poor | | |
| 1. How do you think your mental well-being was: | | | | |
| 1. Before you knew you were pregnant? | | 1. After you found out you were pregnant? | | |
| □ Very good  □ Neither well nor poor  □ Sort of poor □ Very poor | | □ Very good  □ Neither well nor poor  □ Sort of poor □ Very poor | | |

| 1. Do you want to have a child (more children) in the future? | | |
| --- | --- | --- |
| No | Don’t know | Yes |
| 1. Is there anything else you would like to add?   ...............................................................................................................................................................................  ...............................................................................................................................................................................  ..............................................................................................................................................................................  ................................................................................................................................................................................ | | |

FINALLY – THANKS FOR ANSWERING THE QUESTIONS!
